# An excellent navigation system and experience in craniomaxillofacial navigation surgery: a double-center study

**DOI:** 10.1038/srep28242

**Published:** 2016-06-16

**Authors:** Jiewen Dai, Jinyang Wu, Xudong Wang, Xudong Yang, Yunong Wu, Bing Xu, Jun Shi, Hongbo Yu, Min Cai, Wenbin Zhang, Lei Zhang, Hao Sun, Guofang Shen, Shilei Zhang

**Affiliations:** 1 Department of Oral & Cranio-maxillofacial Surgery, Shanghai Ninth People’s Hospital, Shanghai Jiao Tong University School of Medicine, Shanghai Key Laboratory of Stomatology, No. 639 Zhizaoju Road, Shanghai 200011, China; 2 Department of Oral and maxillofacial surgery, Nanjing Stomatological Hospital, Nanjing University Medical School, Nanjing China; 3 Department of Oral and maxillofacial surgery, Affiliated Stomatological Hospital of Nanjing Medical University, Nanjing China

## Abstract

Numerous problems regarding craniomaxillofacial navigation surgery are not well understood. In this study, we performed a double-center clinical study to quantitatively evaluate the characteristics of our navigation system and experience in craniomaxillofacial navigation surgery. Fifty-six patients with craniomaxillofacial disease were included and randomly divided into experimental (using our AccuNavi-A system) and control (using Strker system) groups to compare the surgical effects. The results revealed that the average pre-operative planning time was 32.32 mins *vs* 29.74 mins between the experimental and control group, respectively (p > 0.05). The average operative time was 295.61 mins *vs* 233.56 mins (p > 0.05). The point registration orientation accuracy was 0.83 mm *vs* 0.92 mm. The maximal average preoperative navigation orientation accuracy was 1.03 mm *vs* 1.17 mm. The maximal average persistent navigation orientation accuracy was 1.15 mm *vs* 0.09 mm. The maximal average navigation orientation accuracy after registration recovery was 1.15 mm *vs* 1.39 mm between the experimental and control group. All patients healed, and their function and profile improved. These findings demonstrate that although surgeons should consider the patients’ time and monetary costs, our qualified navigation surgery system and experience could offer an accurate guide during a variety of craniomaxillofacial surgeries.

Trauma, tumor, and developmental malformation lead to severe defects or deformities in the oral and craniomaxillofacial region. Precise reconstruction or correction of those defects/deformities remains a surgical challenge. With the development of modern digital surgery technology, computer-assisted simulation and navigation (CASN) has been described as a useful strategy for clinical application^1^,^2^,^3^,^4^. In oral and craniomaxillofacial surgery, navigation technology has been reported in numerous applications, including removal of foreign bodies, bony tumor resection, reduction of the fractures, deformity correction, defect reconstruction and gap arthroplasty for the temporomandibular joint ankylosis^1^,^5^,^6^,^7^,^8^. Although numerous previous retrospective case series studies indicate that this new method could obtain safe, precise, and effective clinical results, whether the protocol results in improved outcomes compared with the traditional method and whether the actual surgical outcomes are the same as the planned outcomes have not been systematically and quantitatively studied^8^,^9^,^10^. In addition, the efficacy or shortcomings of this navigation protocol have not been conclusively determined. In our department, we have been using CASN technology since 2003, and several articles were published to introduce this method^1^,^5^,^11^,^12^,^13^,^14^,^15^,^16^,^17^. In this study, the authors completed a prospective, double center clinical study to systematically and quantitatively evaluate the excellence and shortcomings of our CASN system and protocols in oral and craniomaxillofacial surgery to help surgeons and patients to choose suitable surgical options.

## Results

### Operative times for navigation surgery

The preoperative computed tomography (CT) images of the craniomaxillofacial region were stored in a Digital Imaging and Communications (DICOM) formatand were imported into the navigation system for preoperative planning, including target image segmentation, three-dimensional (3D) skull model reconstruction, mirror, and surgery simulation. The total pre-operative planning times are presented in Supplementary Table S1. The average pre-operative planning time was 32.32 mins *vs* 29.74 mins in the experimental and control groups, respectively (p > 0.05).

After preparation of the navigation equipment during surgery, registration between the patient and a 3D virtual model and the surgical navigation was performed. The total navigational operative times are presented in Table 1. The average operative times were 295.61 mins *vs* 233.56 mins in the experimental and control groups, respectively (p > 0.05).

### Navigation orientation accuracy

Our previous retrospective studies showed that the mean deviation between the preoperative design and actual surgical results was 1.46 mm based on navigation surgery in the craniomaxillofacial region. In this study, we systematically evaluated the accuracy of navigation surgery from several aspects. The registration orientation accuracy for the point registration method, a frequently used method for navigation surgery,was 0.83 mm *vs* 0.92 mm in the experimental and control groups in this study, respectively (Table 2). The preoperative navigation orientation accuracy of eight different anatomical points is presented in Table 3, and these results demonstrated that the maximal average preoperative navigation orientation accuracy was 1.03 mm *vs* 1.17 mm in the experimental and control groups, respectively, for all eight points. In addition, the minimum average preoperative navigation orientation accuracy existed in point one in both the experimental and control groups. Subsequently, we evaluated the intraoperative navigation orientation accuracy, and the results showed that it was similar to the preoperative navigation orientation accuracy (Table 4). Then, we measured the persistence of navigation orientation accuracy during surgery, and the results revealed that the maximal average persistent navigation orientation accuracy was 1.15 mm *vs* 0.09 mm in the experimental and control groups, respectively, for all eight points (Table 5). Finally, we tested the navigation orientation accuracy after registration recovery (NOA-RR indicates the accuracy between target points in patient and 3D virtual model after re-registration between the patient and 3D virtual model due to the displacement of the digital reference frame during surgery), and the results showed that the maximal average navigation orientation accuracy after registration recovery was 1.15 mm *vs* 1.39 mm in the experimental and control groups, respectively, for all four points (Supplementary Table S2).

### Effect of navigation surgery

Reduction of fractured bone, orbital floor reconstruction, craniomaxillofacial recontouring, TMJ arthroplasty, tumor resection, mandibular osteo-distraction and foreign body removal based on navigation surgery were performed successfully in 55 patients, with the exception of one patient who dropped out due to heavy hemorrhaging during surgery. All patients healed,and their function and profile obviously improved. No serious complications occurred among all patients. Using patients with facial asymmetry as an example, facial asymmetry, which was evaluated by 3dMD photogrammetric measurement combined with CT and clinical examination, improved significantly based on navigation jaw recontouring after 6 months of follow-up (Table 6).

## Discussion

In this double center clinical trial, we systematically evaluated the efficacy of our navigation system and experience in craniomaxillofacial navigation surgery. Previous studies showed that image-guided navigation had many potential applications in oral and craniomaxillofacial surgery, including localization of pathological lesions or foreign bodies, fracture reduction, gap arthroplasty for the temporomandibular joint ankylosis, orthognathic surgery, jaw tumor resection, and surgical corrections of craniomaxillofacial malformations or facial asymmetries^1^,^2^,^11^,^12^,^18^,^19^. In this study, we systematically confirmed these indications and mandible distraction osteogenesis. Numerous navigation systems have been widely reported, and within the craniofacial region, most hardware and software appear to be more suited to neurology, ear, nose, and throat surgery. The AccuNavi-A system consists of a software workstation that is special for craniofacial surgery and an optical navigation platform. More diverse functions can be applied in the special software platform for craniofacial surgery, such as mirroring, measuring and simulating, and we can add personalized functions when necessary. In addition, surgical plans, which were completed with other surgical simulation software, can be input into this AccuNavi-A system via external STL format data and aligned during the preoperative planning. Registration methods, including point registration, surface registration and hybrid registration, can be applied easily and quickly. Surgical plans can be modified intraoperatively according to clinical demands. Furthermore, mandibular navigation surgery is available with our smaller specifically designed dynamic reference frame. Taking these advantages together, we believe our navigation system and experience could be a useful choice for these craniofacial diseases.

Most patients enrolled in our studies were middle age to young people. One possible reason for this age group is that most patients with these diseases were among that age. Of course, we should also realize that these individuals have good health conditions to tolerate the surgery process. As the results showed, the surgery times for navigation surgery are sustained from 50 mins to 681 mins (average 295.61 mins in control group), indicating significant time costs for patients and surgeons and that patients must be in good health to tolerate the surgery. In addition, an experienced doctor is necessary for virtual surgery design, and a preoperative virtual planning time was also required for patients. In theory,any condition that meets the basic technique and physical requirements for navigation surgery could be a candidate for navigation surgery. However, use of the navigation system during surgery plan and surgery procedure requires more time and is cost higher than routine surgery procedures. Thus, surgeons should consider the time and the increased monetary costs for using the navigation system and the anesthetization time requirements. The purpose for navigation surgery is to improve accuracy and safety and to decrease surgical risk. We suggested that CASN be mainly applied to these patients with complicated disease conditionsor patients who require very high accuracy that necessitates a detailed preoperative virtual planning and intraoperative real-time navigation, such as complex zygomaxillary and orbital wall fractures, midfacial comminuted fractures, temporomandibular joint ankylosis, recontouring of craniofacial fibrous dysplasia, recontouring of mandibular angle hypertrophia, removal of facial foreign bodies and resection of complex cartilage or bone tumors. In contrast, the procedure is not routinely recommended for some simple conditions, such as mandibular linear fracture.

Previous case series studies reported the accuracy of navigation surgery in the craniomaxillofacial region based on the deviation between the preoperative design and actual surgery, which may be influenced, at least partially, by the proficiency of the surgeons^3^,^12^,^14^,^20^. In this study, we systematically evaluated the system accuracy of the navigation system itself from different aspects using two different navigation systems in two surgery centers. Our finds revealed satisfactory registration accuracy that was coincident with previous finds. Additionally, our findings also demonstrated good PRNOA, INOA, PENOA and NOA-RR for the navigation system, which further supported the applications of the qualified navigation surgery system in the craniomaxillofacial region.

This study demonstrated satisfactory outcomes based on navigation surgery for all 55 patients with complicated craniomaxillofacial disease. In particular, our quantitative data revealed that navigation surgery could significantly improve the asymmetry in two navigation surgery groups. These finds combined with previous qualitative findings provide sufficient accuracy for highly precise craniomaxillofacial surgery.

In summary, the qualified navigation surgery system can offer accurate guides during a variety of craniomaxillofacial surgeries. When combined with an excellent surgical team, the system can provide sufficient accuracy to obtain satisfactory outcomes for highly precise craniomaxillofacial surgery. However, craniomaxillofacial navigation surgery requires a long operative time, and surgeons should consider the patients’ health conditions as well as time and monetary costs. Taking these finds together, we suggested that craniomaxillofacial navigation surgery should be primarily applied in patients with complicated disease conditions or those who require highly accurate, detailed preoperative virtual planning and intraoperative real-time navigation.

## Methods

### Study design and participants

This study was performed in the Department of Oral and Craniomaxillofacial Surgery, Ninth People’s Hospital, Shanghai Jiao Tong University School of Medicine, Shanghai, China, and Nanjing Stomatological Hospital, Nanjing University, Nanjing, China from 2011 to 2013. All experimental protocols in this clinical prospective study were performed in accordance with STROBE guidelines and CONSORT guidelines and were approved by the Ethics Committee of Shanghai Ninth People’s Hospital, Shanghai Jiaotong University School of Medicine. This study included two groups: experimental (using AccuNavi-A navigation system, Shanghai, China) and control (using Strker Navigation system, USA). The non-inferiority trial was used to compare the results. In addition, α = 0.025, power = 80%, standard deviation(σ) = 0.6 and the dividing value for clinical significance (δ) = 0.5 were established based on the references and suggestion of clinical specialist. Then, the total number of patients was calculated using PASS11 software based on the formula 
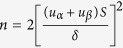
, and a total of 48 patients were necessary in this study. Taking the dropout in clinical practice into consideration, a total of 56 patients diagnosed with zygomatic-orbital-maxillary fractures, jaw tumors, fibrous dysplasia, etc. were enrolled in this study based on clinical need. Informed consent to participate in this study was obtained from all patients, and the patient who appeared in the figure provided full permission for their image to be used in publications in all formats. These 56 patients were allocated at random into the experimental and control groups. The participants and investigators were not aware of allocation, and an independent data manager was enrolled to analyze the obtained data. The distribution of patients among different diseases is presented in Supplementary Table S3. The essential information of these 56 patientsis presented in Supplementary Table S4, and no significant difference in age (average 31.93 and 33.48 years old in the experimental and control groups, respectively), number of registration points (6 to 8 points), and time of registration (average 15.93 and 15.74 minutes in the experimental and control groups, respectively) were noted. The distribution of patients in different centers is presented in Supplementary Table S5, and a patient in the control group dropped out due to heavy hemorrhaging during surgery.

### Surgical navigation procedure

**Figure 1:**
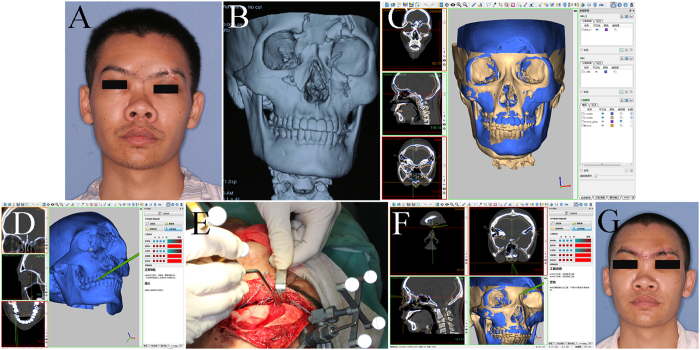
The navigation process. (**A**) A preoperative photograph of patient. (**B**) Patients’ preoperative 3D craniomaxillofacial CT image. (**C**) Preoperative planning and surgery simulation during navigation system. (**D**) Registration between the patient and the 3D virtual craniomaxillofacial model. (**E**,**F**) Surgeons performed the surgery according to the preoperative plan based on the guidance of the navigation system. (**G**) A postoperative photograph of the patient.

The Stryker navigation system (Kalamazoo, USA), a system has been wildly applied in craniomaxillofacial surgery worldwide, was used in the control group in this study, whereas the AccuNavi-A navigation system, a navigation system developed by our team, was used in the experimental group. The navigation protocol for patients with craniomaxillofacial disease has been described in detail in our previous studies. Briefly, patients’ preoperative craniomaxillofacial CT images were imported into the navigation system for preoperative planning and surgery simulation, and then registration between the patient and 3D virtual craniomaxillofacial model using a point-to-point registration method was completed during surgery (Supplementary Fig. S1). Subsequently, the surgeons could perform the surgery according to the preoperative planning based on the guidance of the navigation system. A typical patient was chosen to demonstrate this process and is presented in Fig. 1.

### Statistics

We performed a statistical assessment of the basic information of patients. We used the exact probability calculation for comparison enumeration data between the two groups and the group t test for comparison of measurement data between the two groups. The Wilcoxon rank-sum test was used for comparison of a number of registration points and time of registration between the two groups.

To compare the pre-operative planning times between two navigation surgery groups, we used Wilcoxon rank-sum test for statistics.

To compare the total navigational operative times between two navigation surgery groups, we used the group t test for statistics.

To compare the registration orientation accuracy (ROA) between the two navigation surgery groups, we used the confidence interval (CI) method for the non-inferiority trial, establishing α = 0.025 and δ = 0.5. If the upper limit of the 95% CI of the D-value of ROA C_U_ < 0.5, the ROA of the experimental group was non-inferior compared with the control group. ROA indicates the accuracy of registration points between the patient and 3D virtual model.

**Figure 2:**
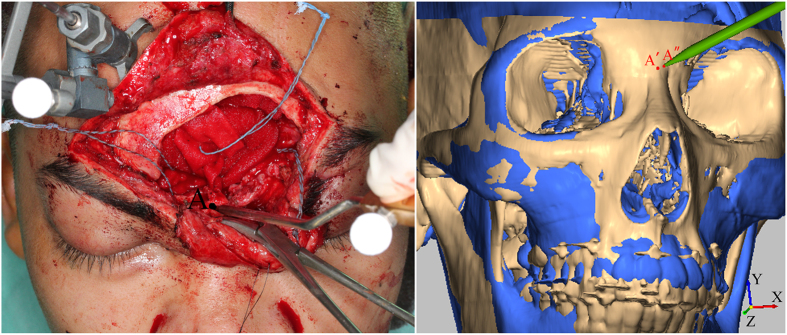
Definition of the meaning and computational formula of several navigation accuracy systems. Point “A” on the patient theoretically corresponded to A′ in the 3D virtual skull model during the navigation surgery; however, this point may actually be displayed as A″ due to deviation between the patient and the navigation system. The preoperative/intraoperative navigation orientation accuracy was defined as the accuracy between the target points in the patient and the 3D virtual model before or during surgery, and the computational formulas for the model before and after surgery were 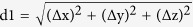, and 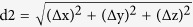, respectively. The persistence of navigation orientation accuracy was defined as the continual accuracy between the target points in the patient and the 3D virtual model during the entire surgery process, and the computational formula was 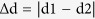. Δx, Δy and Δz indicate the difference value of three-dimensional coordinates between A′ and A″.

To compare the preoperative/intraoperative navigation orientation accuracy (PRNOA/INOA) between two navigation surgery groups, we used the group t test for statistics. PRNOA/INOA indicates the accuracy between the target points in the patient and the 3D virtual model before or during surgery (Fig. 2), and eight points were chosen for every patient.

To compare the persistence of navigation orientation accuracy (PENOA) between two navigation surgery groups, we use the Wilcoxon rank-sum test for statistics. PENOA indicates the continual accuracy between the target points in the patient and the 3D virtual model during the entire surgery process (Fig. 2), and eight points were chosen for every patient.

To compare the improvement of asymmetry between pre- and post-operation within the same group or between two navigation surgery groups, we used the Wilcoxon Sign Rank Test for comparison between pre- and post-operation within the same group and the Wilcoxon rank-sum test for comparison of reduced value between experimental and control groups.

The comprehensive performance of the Stryker navigation system and the AccuNavi-A navigation system were also compared to confirm that both navigation systems were effective and could be applied in this study (data not show).

All statistical analyses were performed using SAS9.2 software, and the measurement data were presented as the mean and standard deviation (SD) values. p < 0.05 was considered statistically significant.

## Additional Information

**How to cite this article**: Dai, J. *et al*. An excellent navigation system and experience in craniomaxillofacial navigation surgery: a double-center study. *Sci. Rep.* **6**, 28242; doi: 10.1038/srep28242 (2016).

## Supplementary Material

Supplementary Information

## Figures and Tables

**Table 1:** Comparison of total navigational operative times (minutes) between two navigation surgery groups.

Group	Number of patients	Mean	Standard deviation	Minimum	P25	Median	P75	Maximum	Statistical magnitude(t)	P-value
Experimental	28	295.61	167.30	60.00	160.00	276.00	415.00	681.00	1.6734	0.1001
Control	27	233.56	97.27	50.00	165.00	220.00	326.00	405.00		

Using the group t test for statistics; the statistical magnitude was t.

**Table 2:** Comparison of registration orientation accuracy (mm) between two navigation surgery groups and non-inferiority trial.

Group	Number of patients	Mean	Standard deviation	Minimum	P25	Median	P75	Maximum	95% CI of D-value of ROA	Statistical conclusion
Experimental	28	0.83	0.11	0.64	0.80	0.82	0.91	1.05	(−0.1597–0.0203)	ROA of EG was non-inferior to CG
Control	27	0.92	0.15	0.68	0.82	0.92	1.00	1.33		

Using the confidence interval method for the n**on-inferiority trial**, α = 0.025, δ = 0.5. If the upper limit of the 95% CI of the D-value of ROAC_U_ < 0.5, the ROA of EG was non-inferior compared with CG.CI: confidence interval; D-value: difference value; ROA: registration **orientation** accuracy; EG: experimental group; CG: control group.

**Table 3:** Comparison of preoperative navigation orientation accuracy (mm) between two navigation surgery groups.

Point	Group	Number of patients	Mean	Standard deviation	Minimum	P25	Median	P75	Maximum	Statistical magnitude (t)	P-value
1	Experimental	28	0.65	0.12	0.43	0.55	0.61	0.73	0.96	2.1360	0.0373
	Control	27	0.73	0.16	0.51	0.58	0.72	0.82	1.11		
2	Experimental	28	0.96	0.25	0.57	0.75	0.92	1.20	1.34	1.1433	0.2581
	Control	27	1.03	0.22	0.70	0.88	1.02	1.17	1.45		
3	Experimental	28	1.02	0.22	0.44	0.90	0.99	1.12	1.50	2.3543	0.0223
	Control	27	1.16	0.23	0.61	1.03	1.16	1.31	1.58		
4	Experimental	28	1.03	0.27	0.55	0.87	1.00	1.27	1.50	1.5189	0.1347
	Control	27	1.14	0.24	0.64	0.95	1.11	1.31	1.62		
5	Experimental	28	1.02	0.26	0.56	0.83	1.03	1.18	1.47	0.7573	0.4522
	Control	27	1.07	0.20	0.68	0.93	1.09	1.22	1.40		
6	Experimental	28	1.02	0.26	0.56	0.76	1.06	1.20	1.52	2.0873	0.0417
	Control	27	1.15	0.23	0.72	0.99	1.19	1.30	1.63		
7	Experimental	28	1.01	0.34	0.52	0.72	1.01	1.28	1.81	1.5953	0.1166
	Control	27	1.13	0.21	0.72	1.01	1.13	1.23	1.65		
8	Experimental	28	1.02	0.23	0.58	0.82	1.01	1.20	1.46	2.3146	0.0245
	Control	27	1.17	0.24	0.80	0.93	1.17	1.33	1.60		

Using the group t test for statistics; the statistical magnitude was t.

**Table 4:** Comparison of intraoperative navigation orientation accuracy (mm) between two navigation surgery groups.

Point	Group	Number of patients	Mean	Standard deviation	Minimum	P25	Median	P75	Maximum	Statistical magnitude (t)	P-value
1	Experimental	28	0.66	0.14	0.45	0.57	0.63	0.77	0.92	2.1772	0.0339
	Control	27	0.75	0.16	0.48	0.60	0.75	0.83	1.16		
2	Experimental	28	0.99	0.25	0.60	0.80	0.91	1.21	1.44	0.5917	0.5566
	Control	27	1.03	0.22	0.65	0.85	0.98	1.22	1.53		
3	Experimental	28	1.03	0.21	0.36	0.95	1.04	1.14	1.37	2.5465	0.0138
	Control	27	1.18	0.22	0.72	1.06	1.16	1.34	1.68		
4	Experimental	28	1.02	0.25	0.51	0.84	1.04	1.22	1.44	1.8726	0.0666
	Control	27	1.14	0.23	0.66	0.96	1.13	1.25	1.68		
5	Experimental	28	1.01	0.25	0.50	0.86	1.01	1.18	1.47	1.6560	0.1036
	Control	27	1.11	0.19	0.78	1.00	1.07	1.26	1.51		
6	Experimental	28	1.06	0.24	0.64	0.88	1.05	1.21	1.61	1.2265	0.2254
	Control	27	1.14	0.23	0.65	0.98	1.11	1.33	1.58		
7	Experimental	28	1.04	0.27	0.60	0.78	1.00	1.28	1.52	1.0343	0.3057
	Control	27	1.11	0.21	0.75	0.96	1.11	1.28	1.57		
8	Experimental	28	1.03	0.24	0.68	0.85	1.05	1.20	1.48	2.0347	0.0469
	Control	27	1.15	0.20	0.74	1.03	1.17	1.33	1.52		

Using the group t test for statistics; the statistical magnitude was t.

**Table 5:** Comparison of persistence of navigation orientation accuracy (mm) between two navigation surgery groups.

Point	Group	Number of patients	Mean	Standard deviation	Minimum	P25	Median	P75	Maximum	Statistical magnitude (Z)	P-value
1	Experimental	28	0.08	0.03	0.03	0.06	0.08	0.10	0.14	0.7874	0.4310
	Control	27	0.09	0.03	0.03	0.06	0.09	0.11	0.15		
2	Experimental	28	0.10	0.09	0.02	0.05	0.10	0.12	0.52	0.4739	0.6355
	Control	27	0.08	0.03	0.03	0.06	0.08	0.10	0.13		
3	Experimental	28	0.09	0.08	0.02	0.06	0.07	0.12	0.45	0.3556	0.7221
	Control	27	0.08	0.03	0.03	0.05	0.09	0.12	0.13		
4	Experimental	28	0.09	0.08	0.02	0.07	0.08	0.10	0.47	1.2323	0.2179
	Control	27	0.09	0.03	0.02	0.07	0.09	0.10	0.14		
5	Experimental	28	0.10	0.09	0.04	0.06	0.08	0.10	0.52	1.4331	0.1518
	Control	27	0.09	0.03	0.02	0.07	0.09	0.11	0.15		
6	Experimental	28	0.08	0.03	0.00	0.07	0.09	0.10	0.14	0.8061	0.4202
	Control	27	0.08	0.03	0.03	0.06	0.08	0.10	0.12		
7	Experimental	28	0.15	0.17	0.05	0.07	0.10	0.11	0.71	1.1833	0.2367
	Control	27	0.08	0.04	0.01	0.06	0.09	0.12	0.14		
8	Experimental	28	0.09	0.06	0.00	0.06	0.08	0.12	0.30	0.4738	0.6357
	Control	27	0.09	0.03	0.04	0.07	0.09	0.12	0.14		

Using the Wilcoxon rank-sum test for statistics; the statistical magnitude was Z.

**Table 6:** Comparison of improvement of asymmetry (%) between pre- and post-operation within the same group or between two navigation surgery groups.

Group	Preoperative	Postoperative	Reduced value	Comparison between pre-and post-operation within the same groups		Comparison reduced value between experimental and control groups	
				Statistical magnitude (**S**)	**P-value**	**Statistical magnitude** (**Z**)	**P-value**
Experimental	10.41 ± 9.21(27); 7.30(2.90~43.80)	2.87 ± 3.67(27); 1.50(0.00~15.60)	7.54 ± 8.24(27); 5.50(−1.39~42.60)	187.00	0.0000	2.5502	0.0108
Control	11.56 ± 7.78(23); 10.20(2.40~40.00)	1.55 ± 2.25(23); 0.90(0.00~10.00)	10.01 ± 6.02(23); 8.30(1.80~30.00)	138.00	0.0000		

Preoperative, postoperative and reduced values were presented as the mean ± SD, Median (Minimum ~ Maximum). Using the Wilcoxon Sign Rank Test for comparison between pre- and post-operation within the same group; the statistical magnitude was S. Using the Wilcoxon rank-sum test for comparisons of reduced values between the experimental and control groups; the statistical magnitude was Z.
