# Supplementary material for: An excellent navigation system and experience in craniomaxillofacial navigation surgery: a double-center study
Source: Sci Rep. 2016 Jun 16;6:28242. doi: 10.1038/srep28242 (PMC4910165; doi:10.1038/srep28242)
Supplement: Supplementary Information [file srep28242-s1.pdf]

An excellent navigation system and experience in craniomaxillofacial navigation surgery: a double-center study

Jiewen Dai, Jinyang Wu, Xudong Wang, Xudong Yang, Yunong Wu, Bing Xu, Jun Shi, Hongbo Yu, Min Cai, Wenbin Zhang, Lei Zhang, Hao Sun, Guofang Shen, Shilei Zhang

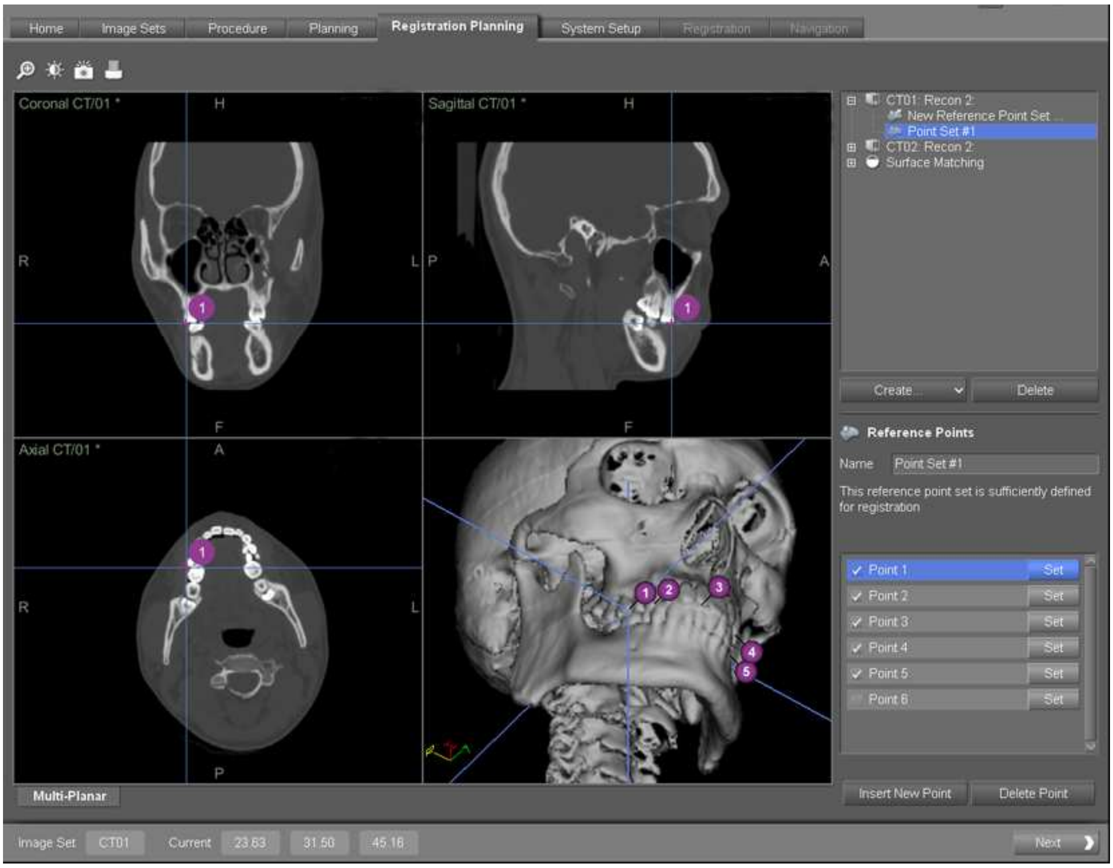

Supplementary Figure S1. Registration between the patient and the 3D virtual craniomaxillofacial model was based on several anatomical points on the teeth; this registration was completed during surgery.

Supplementary Table S1.Comparison of pre-operative planning times (minutes) between two navigation surgery groups

Group	Number of patients	Mean	Standard deviation	Minimum	P25	Median	P75	Maximum	Statistical magnitude (Z)	P-value
Experimental	28	32.32	14.69	10.00	25.00	30.00	42.50	70.00	0.6145	0.5389
Control	27	29.74	11.30	15.00	20.00	25.00	40.00	60.00		

Using Wilcoxon rank-sum test for statistics. Statistical magnitude was Z.

Supplementary Table S2. Comparison of navigation orientation accuracy (mm) after registration recovery between two navigation surgery groups

Point	Group	Number of patients	Mean	Standard deviation	Minimum	P25	Median	P75	Maximum
1	Experimental	3	0.72	0.21	0.51	0.51	0.74	0.92	0.92
	Control	5	0.85	0.17	0.66	0.78	0.83	0.85	1.13
2	Experimental	3	0.88	0.22	0.68	0.68	0.84	1.11	1.11
	Control	5	1.27	0.21	1.03	1.07	1.36	1.42	1.47
3	Experimental	3	1.15	0.34	0.77	0.77	1.26	1.43	1.43
	Control	5	1.31	0.25	0.94	1.21	1.37	1.50	1.55
4	Experimental	3	1.08	0.37	0.74	0.74	1.03	1.48	1.48
	Control	5	1.39	0.16	1.22	1.31	1.36	1.40	1.64

Supplementary Table S3. Patient diagnosis

Disease/Treatment	Number of patients
Zygomatic-orbital-maxillary fractures	28
Jaw tumor	7
Fibrous dysplasia	7
TMJ ankylosis	4
Foreign body	4
Jaw contouring	4
Lefort I and BSSRO	1
Mandibular distraction osteogenesis	1
Total	56

Supplementary Table S4. Essential preoperative patient information

Items		Experimental	Control	Comparison between two groups	
				Statistical magnitude	P-value
Gender	Male	18(64.29%)	19(70.37%)		0.7753
	Female	10(35.71%)	8(29.63%)		
Age (Year)		31.93±11.07(28)	33.48±10.92(27)	0.5237	0.6026
Systolic pressure (mmHg)		108.71±19.55(28)	111.19±15.46(27)	0.5187	0.6061
Diastolic pressure (mmHg)		68.36±13.43(28)	68.89±11.39(27)	0.1581	0.8750
Resting heart rate (Times/minutes)		68.96±13.09(28)	69.37±12.34(27)	0.1183	0.9063
Navigation orientation	Surface registration	1(3.57%)	0(0.00%)		1.0000
	Point registration	27(96.43%)	27(100.00%)		
Number of registration points		6.14±0.97(28)	6.44±1.15(27)	0.9096	0.3630
Time of registration		15.93±4.43(28)	15.74±4.06(27)	0.0256	0.9796

Using the exact probability calculation for comparison enumeration data between the two groups.

Using a group t test for comparison measurement data between the two groups, statistical magnitude was t.

Using a Wilcoxon rank-sum test for comparison of the number of registration points and time of registration between the two groups, statistical magnitude was Z.

Supplementary Table S5. Patient distribution in different centers

Center	Enrolled cases			Dropout cases			Excluded cases			Completed cases		
	Experimental	Control	Total	Experimental	Control	Total	Experimental	Control	Total	Experimental	Control	Total
Shanghai Ninth People's Hospital	16	16	32	0	1	1	0	0	0	16	15	31
Nanjing Stomatological Hospital	12	12	24	0	0	0	0	0	0	12	12	24
Total	28	28	56	0	1	1	0	0	0	28	27	55

Enrolled cases: 56; Dropout cases: 1 (Case 3 in the control group dropped out due to heavy hemorrhage); Dropout rate: 1.79%; Excluded cases: 0; Excluded rate: 0.00%; Completed cases: 55
